# Supplementary material for: Evaluation of Recurrence Risk in Irreversible Electroporation-Treated Pancreatic Adenocarcinoma Patients Using Radiomics Signatures
Source: Cancers (Basel). 2025 Jul 15;17(14):2338. doi: 10.3390/cancers17142338 (PMC12293390; doi:10.3390/cancers17142338)
Supplement: Supplementary file 1 [file cancers-17-02338-s001.zip › cancers-3661071-supplementary.pdf]

Supplementary Materials S1

S1.1 Hand Selected Radiomics Features

A select subset of four clinically motivated radiomics features were selected by us and individually examined to identify any existing correlations with survival endpoints. These features are the mean, 10th percentile, and 90th percentile of the ROI's density histogram, as well as the GLCM sum entropy. GLCM sum entropy was selected due to previous work on PDAC cohorts which found it meaningfully correlates with survival outcomes (12). The intensity features were selected because they can quantify how the IRE-treated ROI was changed in the 12-week follow-up scans, and especially when utilizing delta radiomics features. We hypothesized that those quantified changes might correlate with a patient's response to IRE therapy.

S1.2 Principal Component Analysis, Composite Radiomics Features

PCA was applied to groups of radiomics features based on their type to offer improved resolution on the type of radiomics features relevant to this problem. As outlined in Section 2.1.4, these groups were shape (16), intensity (18), texture (68), local texture (86) and filter (1892) features. From each of these groups, the top two composite features were chosen and examined to identify any correlations with survival outcomes. Additionally, the top two composite features from PCA applied to the full set of 2078 features were examined for any correlation with survival outcomes. Each composite feature is a linear combination of the radiomics features the PCA was applied to. We refer to these as composite radiomics (CR) features. For example, when PCA is applied to the 16 shape radiomics features, we generate two CR features, shape CR feature 1 and shape CR feature 2. Each of these features is a different linear combination of the 16-component shape radiomics features.

Two CR features are selected to balance the amount of each feature group's total variance represented against the number of hypotheses tested. Two CR features represent over 50% of the variance for each feature group.

Supplementary Materials S2

Table S1 – Selected radiomics features, complete results

Endpoint	Feature Source	Feature	Mean	STD	Mann-Whitney U p-value	AUC	Optimal Threshold	Log Rank p-value	Hazard Ratio	Concordance Index	Median Survival Difference [months]
12m TTR	pre	10th Percentile Intensity	19.48	24.53	0.774	0.47	-4.17	0.131	1.88	0.54	2.43
		Mean Intensity	62.93	31.96	0.103	0.36	52.57	0.102	1.71	0.56	2.99
		90th Percentile Intensity	105.43	70.86	0.062	0.34	78.00	0.029	2.14	0.58	2.89
		GLCM Sum Entropy	3.11	0.44	0.005	0.26	3.23	0.005	2.65	0.65	6.61
	12w	10th Percentile Intensity	10.18	19.36	0.182	0.62	16.00	0.130	1.65	0.57	2.99
		Mean Intensity	54.12	16.76	0.508	0.56	51.39	0.415	1.30	0.53	2.66
		90th Percentile Intensity	96.91	20.22	0.501	0.56	107.17	0.279	1.46	0.54	3.58
		GLCM Sum Entropy	3.26	0.33	0.724	0.47	3.40	0.505	1.27	0.52	5.16
	delta	10th Percentile Intensity	-10.01	23.05	0.481	0.56	-6.45	0.063	1.87	0.56	2.99
		Mean Intensity	-9.64	29.76	0.078	0.65	7.56	0.020	2.44	0.57	3.81
		90th Percentile Intensity	-10.09	70.15	0.065	0.66	10.00	0.217	1.52	0.56	3.22
		GLCM Sum Entropy	0.14	0.49	0.033	0.69	0.28	0.080	1.78	0.60	3.95
18m TTLR	pre	10th Percentile Intensity	19.54	24.14	0.891	0.49	34.00	0.397	1.52	0.50	3.06
		Mean Intensity	64.08	32.68	0.197	0.37	68.01	0.142	1.87	0.56	4.37
		90th Percentile Intensity	108.15	76.86	0.136	0.36	104.40	0.071	2.09	0.57	4.37
		GLCM Sum Entropy	3.13	0.44	0.098	0.34	3.41	0.045	2.83	0.60	16.77
	12w	10th Percentile Intensity	9.06	18.96	0.378	0.59	15.00	0.050	2.07	0.61	9.83
		Mean Intensity	54.22	16.71	0.354	0.59	73.64	0.028	3.31	0.55	4.73
		90th Percentile Intensity	97.87	21.17	0.553	0.56	115.00	0.158	1.83	0.54	4.01
		GLCM Sum Entropy	3.28	0.34	0.843	0.48	3.40	0.843	1.08	0.51	4.83
	delta	10th Percentile Intensity	-11.41	22.97	0.334	0.60	-6.45	0.018	2.46	0.60	14.01
		Mean Intensity	-10.93	30.94	0.133	0.65	-10.87	0.047	2.20	0.59	13.78
		90th Percentile Intensity	-12.29	76.78	0.169	0.64	-11.83	0.058	2.25	0.57	4.37
		GLCM Sum Entropy	0.14	0.49	0.235	0.62	0.02	0.037	2.27	0.62	9.63
18m TTDR	pre	10th Percentile Intensity	18.50	21.05	0.564	0.44	14.00	0.128	1.75	0.54	1.68
		Mean Intensity	63.02	30.97	0.281	0.39	55.00	0.038	2.16	0.58	4.08
		90th Percentile Intensity	107.26	75.78	0.344	0.41	78.00	0.030	2.30	0.59	3.72
		GLCM Sum Entropy	3.13	0.45	0.121	0.35	3.06	0.172	1.64	0.60	4.37
	12w	10th Percentile Intensity	7.64	18.29	0.315	0.60	27.44	0.639	1.24	0.51	2.37
		Mean Intensity	51.21	15.04	0.712	0.46	39.75	0.993	1.00	0.53	2.89
		90th Percentile Intensity	94.33	20.16	0.506	0.43	84.00	0.721	1.15	0.52	2.66
		GLCM Sum Entropy	3.25	0.35	0.209	0.38	3.18	0.588	1.22	0.52	3.02
	delta	10th Percentile Intensity	-11.78	21.18	0.736	0.53	18.00	0.113	2.42	0.54	2.99
		Mean Intensity	-12.90	27.68	0.702	0.54	-46.30	0.052	5.79	0.55	inf
		90th Percentile Intensity	-14.96	74.28	0.902	0.49	13.11	0.636	0.83	0.47	-2.99
		GLCM Sum Entropy	0.11	0.50	0.702	0.54	0.02	0.021	2.52	0.62	8.91
18m OS	pre	10th Percentile Intensity	19.34	24.34	0.692	0.47	-4.17	0.229	1.66	0.53	1.94
		Mean Intensity	62.51	31.17	0.304	0.41	61.88	0.107	1.75	0.54	1.94
		90th Percentile Intensity	104.40	68.88	0.200	0.39	95.00	0.099	1.74	0.57	4.37
		GLCM Sum Entropy	3.10	0.44	0.078	0.35	2.84	0.020	2.18	0.60	4.08
	12w	10th Percentile Intensity	9.50	19.11	0.222	0.60	13.19	0.062	1.84	0.58	5.52
		Mean Intensity	53.70	16.36	0.280	0.59	72.98	0.063	2.47	0.54	4.01
		90th Percentile Intensity	96.32	19.76	0.492	0.56	108.00	0.372	1.37	0.53	3.58
		GLCM Sum Entropy	3.26	0.33	0.992	0.50	3.68	0.343	0.64	0.48	-3.35
	delta	10th Percentile Intensity	-10.52	23.74	0.347	0.58	17.93	0.095	2.08	0.54	2.66
		Mean Intensity	-9.59	29.15	0.158	0.62	21.70	0.124	2.30	0.54	3.58
		90th Percentile Intensity	-9.55	68.11	0.213	0.61	15.00	0.138	1.69	0.56	3.95
		GLCM Sum Entropy	0.16	0.51	0.198	0.61	0.07	0.054	1.93	0.60	3.72
12m RFS	pre	10th Percentile Intensity	19.34	24.34	0.777	0.48	-4.17	0.229	1.66	0.53	1.94
		Mean Intensity	62.51	31.17	0.096	0.36	52.57	0.125	1.66	0.55	2.70
		90th Percentile Intensity	104.40	68.88	0.060	0.34	78.00	0.024	2.19	0.58	3.22

		GLCM Sum Entropy	3.10	0.44	0.005	0.26	3.23	0.005	2.68	0.65	6.41
	12w	10th Percentile Intensity	9.50	19.11	0.271	0.59	16.00	0.085	1.77	0.58	4.57
		Mean Intensity	53.70	16.36	0.578	0.55	50.39	0.326	1.38	0.55	2.70
		90th Percentile Intensity	96.32	19.76	0.679	0.54	107.17	0.217	1.53	0.55	3.81
		GLCM Sum Entropy	3.26	0.33	0.816	0.48	3.40	0.447	1.32	0.53	10.29
	delta	10th Percentile Intensity	-10.52	23.74	0.625	0.54	-6.45	0.069	1.85	0.56	2.99
		Mean Intensity	-9.59	29.15	0.092	0.64	4.59	0.062	1.89	0.57	3.95
		90th Percentile Intensity	-9.55	68.11	0.083	0.65	10.00	0.177	1.57	0.56	3.72
		GLCM Sum Entropy	0.16	0.51	0.026	0.69	0.28	0.091	1.75	0.60	3.95

Table S1 contains results from all examined selected radiomics features, significant or not. Features highlighted in green pass the threshold for significant, while features highlighted in yellow narrowly fall short of the threshold for significance. Each of the 4 features are generated from the pre-surgical images, the 12-week follow-up images, and a delta of the two (pre/12w/delta in Feature Source column). All these features are compared against all five endpoints. For each of these comparisons, the feature's mean and standard deviation (STD) are given. The AUC from an ROC plot, as well as the associated optimal threshold and Mann-Whitney U p-value are given in the following three columns. The remaining 4 columns, the log rank p-value, the hazard ratio, the concordance index, and the median survival difference (in months), are generated from a survival analysis of the high and low risk groups created by applying the optimal threshold. 12 month binarized time to recurrence = 12m TTR; 18 month binarized time to local recurrence = 18m TTLD; 18 month binarized time to distant recurrence = 18m TTDR; 18 month binarized overall survival = 18m OS; 12 month binarized recurrence free survival = 12m RFS; Gray Level Co-Occurrence Matrix = GLCM.

Table S2 – Composite radiomics features, complete results

Endpoint	Feature Source	Feature	Mean	STD	Mann-Whitney U p-value	AUC	Optimal Threshold	Log Rank p-value	Hazard Ratio	Concordance Index	Median Survival Difference [months]
12m TTR	pre	Full CR Feature 1	0.00	27.51	0.014	0.29	-7.50	0.024	2.10	0.61	5.33
		Full CR Feature 2	0.00	16.32	0.002	0.76	-0.38	0.012	2.27	0.62	6.41
		Shape CR Feature 1	0.00	3.18	0.179	0.38	-0.51	0.424	1.30	0.58	4.11
		Shape CR Feature 2	0.00	1.73	0.378	0.58	1.36	0.301	1.51	0.53	2.66
		Intensity CR Feature 1	0.00	3.13	0.034	0.32	1.54	0.008	3.13	0.62	14.01
		Intensity CR Feature 2	0.00	1.71	0.366	0.42	0.61	0.515	1.31	0.54	3.19
		Texture CR Feature 1	0.00	5.17	0.017	0.29	0.65	0.055	1.92	0.61	5.33
		Texture CR Feature 1	0.00	3.71	0.581	0.55	-0.19	0.383	1.33	0.57	4.11
		Filter CR Feature 1	0.00	26.83	0.014	0.29	-8.08	0.038	1.98	0.60	4.44
		Filter CR Feature 2	0.00	15.80	0.002	0.77	-0.64	0.012	2.27	0.62	6.41
		Local Texture CR Feature 1	0.00	4.70	0.453	0.43	3.76	0.477	1.35	0.52	1.94
		Local Texture CR Feature 2	0.00	3.40	0.123	0.37	1.39	0.009	3.07	0.61	17.85
	12w	Full CR Feature 1	0.00	27.40	1.000	0.50	-12.97	0.164	1.59	0.56	3.22
		Full CR Feature 2	0.00	16.82	0.643	0.46	7.88	0.076	1.91	0.57	4.37
		Shape CR Feature 1	0.00	3.12	0.508	0.56	1.49	0.012	2.56	0.61	6.77
		Shape CR Feature 2	0.00	1.97	0.982	0.50	0.53	0.706	1.15	0.52	2.43
		Intensity CR Feature 1	0.00	2.75	0.930	0.49	1.31	0.869	1.07	0.50	10.29
		Intensity CR Feature 2	0.00	1.98	0.522	0.56	0.22	0.150	1.61	0.56	2.99
		Texture CR Feature 1	0.00	5.12	0.895	0.49	1.97	0.961	0.98	0.50	2.37
		Texture CR Feature 1	0.00	3.82	0.860	0.52	-4.28	0.523	1.36	0.54	4.57
		Filter CR Feature 1	0.00	26.78	1.000	0.50	-13.22	0.099	1.77	0.57	3.22
		Filter CR Feature 2	0.00	16.01	0.724	0.47	6.40	0.137	1.69	0.56	3.48
		Local Texture CR Feature 1	0.00	5.62	0.912	0.51	-0.45	0.780	1.10	0.51	0.99
		Local Texture CR Feature 2	0.00	3.45	0.643	0.46	2.34	0.117	2.00	0.55	4.37
	delta	Full CR Feature 1	0.00	27.30	0.162	0.38	3.53	0.114	1.73	0.60	3.95
		Full CR Feature 2	0.00	15.68	0.260	0.60	11.29	0.001	4.00	0.60	4.08
		Shape CR Feature 1	0.00	3.01	0.345	0.58	2.82	0.451	1.38	0.55	3.78
		Shape CR Feature 2	0.00	1.78	0.937	0.49	1.09	0.397	1.37	0.54	2.70
		Intensity CR Feature 1	0.00	3.09	0.094	0.35	1.04	0.002	5.53	0.61	inf
		Intensity CR Feature 2	0.00	1.64	0.609	0.45	-0.20	0.217	1.51	0.55	2.43
		Texture CR Feature 1	0.00	5.09	0.130	0.37	-2.96	0.032	2.02	0.61	4.24
		Texture CR Feature 1	0.00	3.76	0.419	0.43	-2.21	0.074	2.03	0.58	3.85
		Filter CR Feature 1	0.00	26.54	0.169	0.38	3.23	0.071	1.90	0.61	4.60
		Filter CR Feature 2	0.00	15.08	0.207	0.61	10.38	0.006	3.05	0.58	4.01
		Local Texture CR Feature 1	0.00	5.05	0.900	0.49	-2.37	0.054	1.90	0.60	3.91
		Local Texture CR Feature 2	0.00	3.14	0.125	0.37	0.65	0.120	1.73	0.57	4.57
18m TTLR	pre	Full CR Feature 1	0.00	28.04	0.186	0.37	-9.38	0.159	1.68	0.58	4.60
		Full CR Feature 2	0.00	15.53	0.403	0.58	3.34	0.594	1.22	0.53	1.15
		Shape CR Feature 1	0.00	3.20	0.939	0.51	2.08	0.015	2.72	0.60	5.72
		Shape CR Feature 2	0.00	1.84	0.988	0.50	-2.00	0.895	0.92	0.49	-2.66
		Intensity CR Feature 1	0.00	3.10	0.066	0.32	1.34	0.009	3.77	0.62	16.77
		Intensity CR Feature 2	0.00	1.75	0.939	0.49	1.01	0.697	1.21	0.53	3.75
		Texture CR Feature 1	0.00	5.29	0.149	0.36	4.73	0.154	2.12	0.58	16.27
		Texture CR Feature 1	0.00	3.69	0.843	0.52	-0.44	0.761	1.12	0.54	4.80
		Filter CR Feature 1	0.00	27.33	0.207	0.38	-9.50	0.113	1.78	0.60	5.33
		Filter CR Feature 2	0.00	14.93	0.403	0.58	0.92	0.313	1.45	0.55	2.04
		Local Texture CR Feature 1	0.00	4.69	0.773	0.53	-2.81	0.917	0.96	0.51	-1.15
		Local Texture CR Feature 2	0.00	3.07	0.281	0.61	-2.65	0.017	3.09	0.62	17.85
	12w	Full CR Feature 1	0.00	27.80	0.915	0.51	-14.85	0.036	2.41	0.60	3.88
		Full CR Feature 2	0.00	16.90	0.081	0.33	-1.18	0.463	1.31	0.52	1.81
		Shape CR Feature 1	0.00	3.16	0.438	0.42	-2.66	0.025	2.86	0.56	4.60
		Shape CR Feature 2	0.00	1.93	0.988	0.50	-0.59	0.497	0.77	0.44	-4.83

		Intensity CR Feature 1	0.00	2.80	0.682	0.46	1.74	0.850	0.92	0.49	12.49
		Intensity CR Feature 2	0.00	1.98	0.186	0.37	-1.47	0.153	1.76	0.56	4.24
		Texture CR Feature 1	0.00	5.40	0.595	0.55	-2.37	0.036	2.41	0.60	3.88
		Texture CR Feature 1	0.00	3.78	0.267	0.39	-1.29	0.777	0.90	0.48	-2.66
		Filter CR Feature 1	0.00	27.09	0.964	0.51	-14.80	0.019	2.82	0.61	8.91
		Filter CR Feature 2	0.00	16.18	0.111	0.35	-1.94	0.463	1.31	0.52	1.81
		Local Texture CR Feature 1	0.00	5.62	0.494	0.43	-1.10	0.960	1.02	0.50	-2.04
		Local Texture CR Feature 2	0.00	3.65	0.750	0.47	1.30	0.156	1.74	0.56	8.68
	delta	Full CR Feature 1	0.00	27.64	0.740	0.47	2.49	0.023	2.51	0.62	8.91
		Full CR Feature 2	0.00	16.33	0.716	0.46	10.54	0.119	0.47	0.44	-4.01
		Shape CR Feature 1	0.00	3.06	0.788	0.47	-2.55	0.944	1.03	0.52	1.81
		Shape CR Feature 2	0.00	1.81	0.646	0.45	1.03	0.596	1.25	0.53	2.04
		Intensity CR Feature 1	0.00	3.09	0.496	0.43	2.13	0.058	3.67	0.57	inf
		Intensity CR Feature 2	0.00	1.70	0.117	0.35	0.53	0.559	1.33	0.52	1.15
		Texture CR Feature 1	0.00	5.35	0.248	0.39	-2.58	0.013	2.47	0.64	7.53
		Texture CR Feature 1	0.00	3.82	0.602	0.45	-1.73	0.448	1.34	0.53	1.81
		Filter CR Feature 1	0.00	26.82	0.837	0.48	1.75	0.053	2.10	0.61	5.33
		Filter CR Feature 2	0.00	15.69	0.692	0.46	-12.63	0.160	1.98	0.54	3.58
		Local Texture CR Feature 1	0.00	5.10	0.716	0.46	-1.84	0.309	1.46	0.54	-1.38
		Local Texture CR Feature 2	0.00	3.08	0.837	0.52	0.36	0.663	0.85	0.48	-1.38
18m TTDR	pre	Full CR Feature 1	0.00	27.84	0.143	0.36	2.12	0.054	2.18	0.60	4.37
		Full CR Feature 2	0.00	16.79	0.040	0.70	2.90	0.002	3.02	0.65	6.41
		Shape CR Feature 1	0.00	3.23	0.095	0.34	0.54	0.229	1.55	0.59	8.68
		Shape CR Feature 2	0.00	1.62	0.734	0.47	0.50	0.409	1.40	0.52	1.35
		Intensity CR Feature 1	0.00	3.15	0.188	0.37	1.15	0.011	3.29	0.63	16.77
		Intensity CR Feature 2	0.00	1.76	0.584	0.45	0.70	0.902	0.95	0.50	0.23
		Texture CR Feature 1	0.00	5.18	0.179	0.37	-0.41	0.252	1.52	0.57	4.37
		Texture CR Feature 1	0.00	3.84	0.399	0.42	-1.87	0.224	1.56	0.55	1.35
		Filter CR Feature 1	0.00	27.18	0.143	0.36	1.66	0.054	2.18	0.60	4.37
		Filter CR Feature 2	0.00	16.27	0.032	0.71	-1.94	0.010	2.51	0.64	9.63
		Local Texture CR Feature 1	0.00	4.75	0.367	0.41	-1.19	0.354	1.40	0.54	3.25
		Local Texture CR Feature 2	0.00	3.50	0.988	0.50	1.40	0.122	2.00	0.58	7.40
	12w	Full CR Feature 1	0.00	28.10	0.487	0.43	27.24	0.991	1.00	0.51	11.34
		Full CR Feature 2	0.00	16.46	0.988	0.50	3.73	0.733	1.13	0.50	0.82
		Shape CR Feature 1	0.00	3.09	0.024	0.72	0.25	0.005	2.76	0.64	6.58
		Shape CR Feature 2	0.00	2.02	0.416	0.58	-0.57	0.809	1.09	0.54	1.71
		Intensity CR Feature 1	0.00	2.81	0.294	0.40	2.06	0.829	1.11	0.51	11.34
		Intensity CR Feature 2	0.00	1.92	0.988	0.50	-1.32	0.538	1.27	0.53	2.70
		Texture CR Feature 1	0.00	5.23	0.399	0.42	2.06	0.759	0.89	0.49	1.38
		Texture CR Feature 1	0.00	3.69	0.294	0.60	0.85	0.199	1.64	0.58	4.34
		Filter CR Feature 1	0.00	27.38	0.469	0.43	26.79	0.991	1.00	0.51	11.34
		Filter CR Feature 2	0.00	15.75	0.848	0.52	-14.21	0.357	1.64	0.56	10.49
		Local Texture CR Feature 1	0.00	5.29	0.825	0.52	-3.16	0.846	1.08	0.51	3.29
		Local Texture CR Feature 2	0.00	3.23	0.647	0.45	-1.92	0.728	1.14	0.51	-1.15
	delta	Full CR Feature 1	0.00	27.32	0.818	0.48	18.02	0.046	3.89	0.58	inf
		Full CR Feature 2	0.00	16.25	0.055	0.69	4.28	0.057	1.99	0.60	4.37
		Shape CR Feature 1	0.00	2.99	0.145	0.64	-0.20	0.207	1.58	0.59	3.48
		Shape CR Feature 2	0.00	1.77	0.434	0.42	-1.05	0.459	1.32	0.54	1.71
		Intensity CR Feature 1	0.00	3.08	0.915	0.49	0.77	0.002	7.41	0.62	inf
		Intensity CR Feature 2	0.00	1.65	0.530	0.44	1.34	0.131	2.89	0.55	15.42
		Texture CR Feature 1	0.00	5.07	0.890	0.49	-3.44	0.124	1.77	0.59	4.47
		Texture CR Feature 1	0.00	3.80	0.048	0.31	-0.43	0.008	2.66	0.63	10.78
		Filter CR Feature 1	0.00	26.56	0.818	0.48	8.49	0.039	2.65	0.60	8.68
		Filter CR Feature 2	0.00	15.66	0.041	0.70	9.85	0.000	5.02	0.63	5.98
		Local Texture CR Feature 1	0.00	4.99	0.842	0.52	1.42	0.507	1.29	0.51	1.38
		Local Texture CR Feature 2	0.00	3.06	0.939	0.51	-2.03	0.760	1.14	0.52	-1.38
18m OS	pre	Full CR Feature 1	0.00	27.08	0.218	0.39	-7.18	0.029	2.05	0.60	4.60
		Full CR Feature 2	0.00	16.57	0.152	0.62	5.25	0.004	2.68	0.63	5.72
		Shape CR Feature 1	0.00	3.14	0.355	0.42	0.89	0.239	1.48	0.57	5.16
		Shape CR Feature 2	0.00	1.81	0.815	0.52	1.62	0.930	0.96	0.49	0.69
		Intensity CR Feature 1	0.00	3.07	0.075	0.35	-0.15	0.148	1.63	0.56	2.66

		Intensity CR Feature 2	0.00	1.71	0.344	0.58	-0.77	0.484	1.28	0.52	1.94
		Texture CR Feature 1	0.00	5.13	0.066	0.34	-0.93	0.047	1.92	0.59	4.60
		Texture CR Feature 1	0.00	3.69	0.268	0.59	-1.36	0.821	1.08	0.52	0.69
		Filter CR Feature 1	0.00	26.43	0.250	0.40	-7.56	0.047	1.93	0.60	3.72
		Filter CR Feature 2	0.00	15.94	0.146	0.62	5.23	0.004	2.68	0.63	5.72
		Local Texture CR Feature 1	0.00	4.86	0.692	0.53	2.00	0.629	0.84	0.48	-1.81
		Local Texture CR Feature 2	0.00	3.29	0.387	0.43	0.56	0.071	1.90	0.59	3.88
	12w	Full CR Feature 1	0.00	27.25	0.992	0.50	-26.30	0.912	1.05	0.51	3.75
		Full CR Feature 2	0.00	16.65	0.848	0.48	4.91	0.285	1.45	0.54	2.99
		Shape CR Feature 1	0.00	3.13	0.679	0.46	-2.72	0.061	2.24	0.54	2.66
		Shape CR Feature 2	0.00	1.99	0.473	0.44	-0.50	0.491	0.80	0.44	-2.99
		Intensity CR Feature 1	0.00	2.77	0.816	0.48	0.13	0.833	1.08	0.50	4.96
		Intensity CR Feature 2	0.00	1.97	0.298	0.59	1.61	0.064	1.92	0.57	4.14
		Texture CR Feature 1	0.00	5.13	0.960	0.51	-3.64	0.555	1.24	0.53	-1.81
		Texture CR Feature 1	0.00	3.82	0.565	0.45	-1.24	0.560	1.21	0.52	0.66
		Filter CR Feature 1	0.00	26.62	1.000	0.50	-25.47	0.912	1.05	0.51	3.75
		Filter CR Feature 2	0.00	15.97	0.992	0.50	-20.15	0.352	1.63	0.54	10.49
		Local Texture CR Feature 1	0.00	5.54	0.606	0.54	5.76	0.080	0.30	0.46	-inf
		Local Texture CR Feature 2	0.00	3.35	0.649	0.46	0.27	0.257	1.46	0.53	2.66
	delta	Full CR Feature 1	0.00	27.28	0.746	0.47	9.62	0.048	2.25	0.59	4.37
		Full CR Feature 2	0.00	15.21	0.762	0.53	-7.35	0.602	1.21	0.51	3.75
		Shape CR Feature 1	0.00	3.00	0.908	0.49	-0.86	0.585	0.84	0.48	-2.17
		Shape CR Feature 2	0.00	1.78	0.458	0.56	-0.22	0.864	1.06	0.53	2.14
		Intensity CR Feature 1	0.00	3.03	0.229	0.40	0.56	0.026	2.32	0.59	4.37
		Intensity CR Feature 2	0.00	1.65	0.070	0.34	-0.07	0.948	1.02	0.51	0.66
		Texture CR Feature 1	0.00	5.11	0.433	0.43	2.64	0.207	1.69	0.56	3.19
		Texture CR Feature 1	0.00	3.72	0.992	0.50	1.07	0.657	0.86	0.48	-1.38
		Filter CR Feature 1	0.00	26.54	0.762	0.47	6.86	0.085	1.92	0.59	3.72
		Filter CR Feature 2	0.00	14.63	0.683	0.54	-6.00	0.503	1.26	0.53	3.88
		Local Texture CR Feature 1	0.00	4.96	0.471	0.44	-2.41	0.281	1.46	0.55	3.55
		Local Texture CR Feature 2	0.00	3.03	0.638	0.54	-2.10	0.966	1.01	0.50	-1.81
12m RFS	pre	Full CR Feature 1	0.00	27.08	0.014	0.29	2.56	0.033	2.17	0.61	4.60
		Full CR Feature 2	0.00	16.57	0.012	0.71	5.25	0.004	2.68	0.63	5.72
		Shape CR Feature 1	0.00	3.14	0.193	0.39	-0.50	0.374	1.34	0.58	4.11
		Shape CR Feature 2	0.00	1.81	0.358	0.58	-2.55	0.381	1.69	0.53	13.58
		Intensity CR Feature 1	0.00	3.07	0.040	0.33	1.08	0.011	3.01	0.61	13.78
		Intensity CR Feature 2	0.00	1.71	0.832	0.48	2.15	0.328	2.01	0.53	2.86
		Texture CR Feature 1	0.00	5.13	0.011	0.29	0.80	0.082	1.81	0.60	4.60
		Texture CR Feature 1	0.00	3.69	0.437	0.57	-0.46	0.442	1.29	0.57	4.44
		Filter CR Feature 1	0.00	26.43	0.015	0.29	2.51	0.033	2.17	0.61	4.60
		Filter CR Feature 2	0.00	15.94	0.013	0.71	5.23	0.004	2.68	0.63	5.72
		Local Texture CR Feature 1	0.00	4.86	0.289	0.41	3.34	0.182	1.80	0.54	13.71
		Local Texture CR Feature 2	0.00	3.29	0.077	0.35	0.74	0.026	2.39	0.59	8.38
	12w	Full CR Feature 1	0.00	27.25	0.976	0.50	-12.90	0.120	1.68	0.57	3.72
		Full CR Feature 2	0.00	16.65	0.960	0.51	-22.84	0.352	1.63	0.54	10.49
		Shape CR Feature 1	0.00	3.13	0.565	0.55	1.47	0.013	2.54	0.61	4.47
		Shape CR Feature 2	0.00	1.99	0.635	0.46	0.58	0.494	1.29	0.52	2.14
		Intensity CR Feature 1	0.00	2.77	0.912	0.49	0.13	0.833	1.08	0.50	4.96
		Intensity CR Feature 2	0.00	1.97	0.709	0.53	1.23	0.063	1.89	0.57	4.14
		Texture CR Feature 1	0.00	5.13	0.944	0.49	1.95	0.909	0.96	0.49	2.04
		Texture CR Feature 1	0.00	3.82	0.785	0.52	-3.65	0.719	1.16	0.53	4.27
		Filter CR Feature 1	0.00	26.62	0.960	0.51	-13.13	0.077	1.82	0.58	3.72
		Filter CR Feature 2	0.00	15.97	0.754	0.53	-20.15	0.352	1.63	0.54	10.49
		Local Texture CR Feature 1	0.00	5.54	0.709	0.53	-0.52	0.920	1.04	0.50	2.37
		Local Texture CR Feature 2	0.00	3.35	0.635	0.46	2.59	0.088	2.40	0.56	4.08
	delta	Full CR Feature 1	0.00	27.28	0.164	0.38	4.18	0.125	1.71	0.60	3.72
		Full CR Feature 2	0.00	15.21	0.205	0.61	11.81	0.004	3.21	0.58	4.34
		Shape CR Feature 1	0.00	3.00	0.360	0.58	2.87	0.390	1.44	0.56	4.01
		Shape CR Feature 2	0.00	1.78	1.000	0.50	-2.38	0.768	0.86	0.51	1.81
		Intensity CR Feature 1	0.00	3.03	0.081	0.35	1.03	0.002	5.35	0.60	inf

		Intensity CR Feature 2	0.00	1.65	0.967	0.50	1.18	0.255	1.82	0.54	17.23
		Texture CR Feature 1	0.00	5.11	0.129	0.37	-2.83	0.027	2.07	0.62	4.57
		Texture CR Feature 1	0.00	3.72	0.442	0.43	-2.22	0.053	2.08	0.58	4.41
		Filter CR Feature 1	0.00	26.54	0.164	0.38	3.85	0.077	1.87	0.61	4.44
		Filter CR Feature 2	0.00	14.63	0.190	0.61	-6.00	0.503	1.26	0.53	3.88
		Local Texture CR Feature 1	0.00	4.96	0.934	0.51	3.03	0.747	0.87	0.48	2.14
		Local Texture CR Feature 2	0.00	3.03	0.212	0.39	0.01	0.114	1.72	0.57	4.27

Table S2 contains results from all examined composite radimoics (CR) features, significant or not. Features highlighted in green pass the threshold for significant, while features highlighted in yellow narrowly fall short of the threshold for significance. Each of the 2 CR features from the 6 different/overlapping feature groups, are generated from the pre-surgical images, the 12-week follow-up images, and a delta of the two (pre/12w/delta in Feature Source column). All these features are compared against all five endpoints. For each of these comparisons, the feature's mean and standard deviation (STD) are given. The AUC from an ROC plot, as well as the associated optimal threshold and the associated Mann-Whitney U p-value are given in the following three columns. The remaining 4 columns, the log rank p-value, the hazard ratio, the concordance index, and the median survival difference (in months), are generated from a survival analysis of the high and low risk groups created by applying the optimal threshold. 12 month binarized time to recurrence = 12m TTR; 18 month binarized time to local recurrence = 18m TTLD; 18 month binarized time to distant recurrence = 18m TTDR; 18 month binarized overall survival = 18m OS; 12 month binarized recurrence free survival = 12m RFS.
